# Supplementary material for: Predicting visual function by interpreting a neuronal wiring diagram
Source: Nature. 2024 Oct 2;634(8032):113–23. doi: 10.1038/s41586-024-07953-5 (PMC11446822; doi:10.1038/s41586-024-07953-5)
Supplement: Supplementary file 6 — CRF and ERF predictions for individual TmY4 and TmY9 cells. Analogous to Supplementary Data 3, but for TmY target types. Shown are the top four monosynaptic pathways, the strongest pathway passing through each of the top ten intermediary types (ranking from Extended Data Fig. 7), and the trisynaptic pathway Tm1–TmY–Dm3–TmY (see the section entitled Prediction of spatial normalization). [file 41586_2024_7953_MOESM6_ESM.zip › DataS4/TmY9qΓèÑ/720575940627024134.pdf]

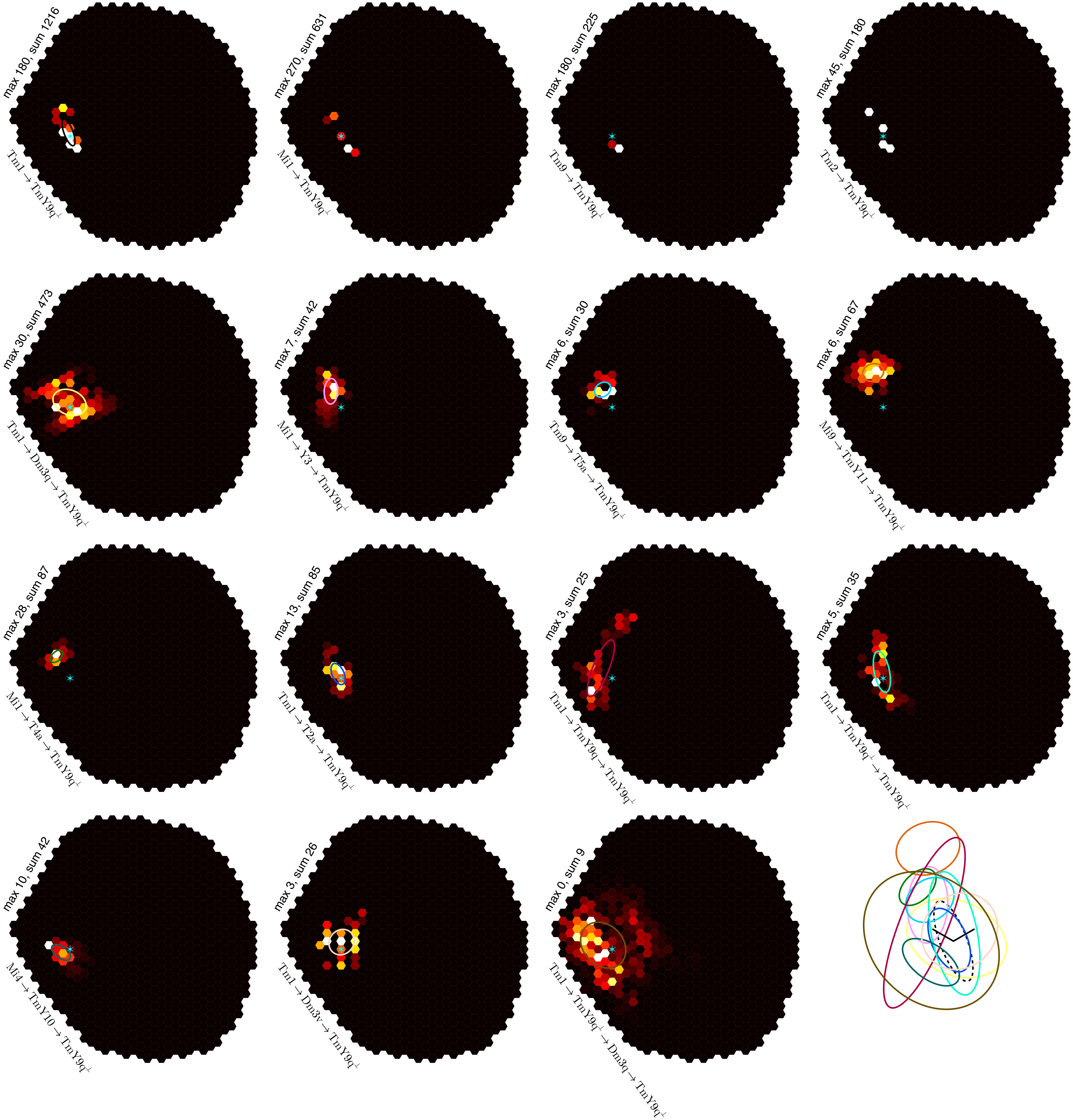

max 180, sum 1216
Tm1 → TmY9q⊥
max 270, sum 631
Mi1 → TmY9q⊥
max 180, sum 225
Tm9 → TmY9q⊥
max 45, sum 180
Tm2 → TmY9q⊥
max 30, sum 473
Tm1 → Dm3q → TmY9q⊥
max 7, sum 42
Mi1 → Y3 → TmY9q⊥
max 6, sum 30
Tm9 → T5a → TmY9q⊥
max 6, sum 67
Mi9 → TmY11 → TmY9q⊥
max 28, sum 87
Mi1 → T4a → TmY9q⊥
max 13, sum 85
Tm1 → T2a → TmY9q⊥
max 3, sum 25
Tm1 → TmY9q → TmY9q⊥
max 5, sum 35
Tm1 → TmY9q⊥ → TmY9q⊥
max 10, sum 42
Mi4 → TmY10 → TmY9q⊥
max 3, sum 26
Tm1 → Dm3v → TmY9q⊥
max 0, sum 9
Tm1 → TmY9q⊥ → Dm3q → TmY9q⊥
